# Machine unlearning: linear filtration for logit-based classifiers

**DOI:** 10.1007/s10994-022-06178-9

**Published:** 2022-07-11

**Authors:** Thomas Baumhauer, Pascal Schöttle, Matthias Zeppelzauer

**Affiliations:** 1 St. Pölten University of Applied Sciences, St. Pölten, Austria; 2 Management Center Innsbruck, Innsbruck, Austria

**Keywords:** Machine learning, Machine unlearning, Privacy

## Abstract

Recently enacted legislation grants individuals certain rights to decide in what fashion their personal data may be used and in particular a “right to be forgotten”. This poses a challenge to machine learning: how to proceed when an individual retracts permission to use data which has been part of the training process of a model? From this question emerges the field of *machine unlearning*, which could be broadly described as the investigation of how to “delete training data from models”. Our work complements this direction of research for the specific setting of class-wide deletion requests for classification models (e.g. deep neural networks). As a first step, we propose *linear filtration* as an intuitive, computationally efficient sanitization method. Our experiments demonstrate benefits in an adversarial setting over naive deletion schemes.

## Introduction

Recently enacted legislation, such as the European Union’s General Data Protection Regulation (GDPR) (Council of European Union 2016), previously its “right to be forgotten” (Council of European Union 2012), and the California Consumer Privacy Act (State of California 2018) grant individuals certain rights to decide in what fashion their personal data may be used, and in particular the right to ask for personal data collected about them to be deleted.

At present the implementation of such rights in the context of machine learning models trained on personal data is largely an open problem. In Villaronga et al. (2018) the authors even conclude that “it may be impossible to fulfill the legal aims of the Right to be Forgotten in artificial intelligence environments”.

Indeed, machine learning models may unintentionally memorize (part of) their training data, leading to privacy issues in many applications, e.g. image classification (Yeom et al. 2018; Sablayrolles et al. 2019) or natural language processing (Carlini et al. 2019), and potentially enabling an adversary to extract sensitive information from a trained model by so-called *model inversion* (Veale et al. 2018).

**Fig. 1:**
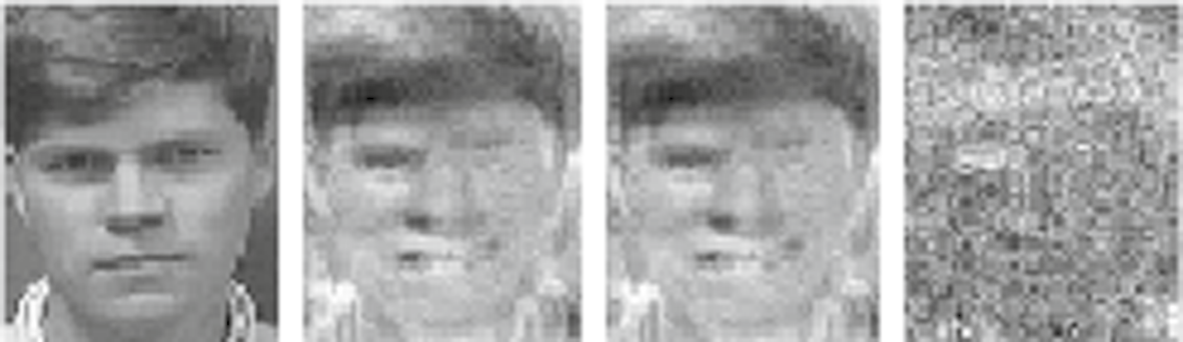
Results of a model inversion attack for a toy model trained on the AT&T Faces dataset with 4 classes. For one of the classes, from left to right: one of the training images, reconstruction of the class by model inversion, reconstruction after naive unlearning, reconstruction after unlearning by our proposed method of *normalizing linear filtration* (defined in Sect. 4). The reconstructions of the other classes remain visually unchanged by normalizing linear filtration, see Fig.5

Informally, deletion of part of the training data from a machine learning model can be understood as removal of its influence on the model parameters, in order to obtain a model that “looks as if it has never seen that part of the data”. We refer to this process as *unlearning*. Clearly, the problem of unlearning can be solved in a trivial fashion, by simply retraining the model without using the part of the data we wish to unlearn. For large, real-world models, retraining from scratch may incur significant computational costs, and may thus be practically infeasible, if deletion requests are expected to arrive at any time. We therefore wish to find more efficient unlearning algorithms, which is notoriously difficult, owing to the fact that for many popular learning algorithms every training data point potentially affects every model parameter.

First approaches towards directed unlearning were introduced in Cao and Yang (2015), and more recently in Ginart et al. (2019); Bourtoule et al. (2019). In our work we consider the problem of unlearning in the setting of classification models for which, in contrast to previous work, we assume that single individuals own all training data associated with a particular class, as may be the case e.g. in biometric applications.

In this setting, we consider classifiers that predict logits, i.e. (rescaled) logarithmic probabilities that a data point belongs to certain classes. For such classifiers we propose a novel sanitization method that applies a linear transformation to these predictions. For an appropriate hypothesis class this transformation can be absorbed into the original classifier. The computation of the transformation requires barely more than computing predictions by the original classifier for a (small) number of data points per class. We call this method *linear filtration*. Figure 1 shows the results of this method when used as a defense against model inversion (Fredrikson et al. 2015).

In summary, the main contributions of our work are:

1. We develop *linear filtration*, a novel algorithm for the sanitization of classification models that predict logits, after class-wide deletion requests.
2. On the theoretical side, we add to the definition of unlearning in the sense of Ginart et al. (2019), by proposing a weakened, “black-box” variant of the definition, which may serve as a more realistic goal in practice.
3. As practical methodology, we suggest that the quality of an empirical unlearning operation may be evaluated in an adversarial setting, i.e. by testing how well it prevents certain privacy attacks on machine learning models.

The rest of this paper is organized as follows: Section 2 gives an overview of work related to machine unlearning, as well the adversarial methodology employed in our experiments. Section 3 formalizes the unlearning of training data from machine learning models. Section 4 establishes several variants of our method of linear filtration. Section 5 experimentally evaluates linear filtration, primarily in an adversarial context. Section 6 features discussion on the definition of unlearning.

## Related work

### Machine unlearning

The term *machine unlearning* first appears in Cao and Yang (2015). There the authors consider unlearning the framework of statistical query learning (Kearns 1998). This allows them to unlearn data points for learning algorithms where all queries to the data are decided upfront. However, many popular learning algorithms (such as gradient descent) query data adaptively. In the adaptive setting the approach of (Cao and Yang 2015) does not give any bounds and quickly falls apart if the number of queries is large, as is the case for neural networks.

Ginart et al. (2019) features a discussion of the problem of efficient unlearning of training data points from models, establishes several engineering principles, and on the practical side proposes unlearning algorithms for *k*-means clustering. In particular, they recognize that given the stochastic nature of many learning algorithms a probabilistic definition of unlearning (there “deletion”) is necessary. We adopt this view in our work.

Bourtoule et al. (2019) propose a framework they refer to as SISA (sharded, isolated, sliced, and aggregated training), which can be thought of as bookkeeping method seeking to limit and keep track of the influence of training data points on model parameters, thus reducing the amount of retraining necessary upon receiving a deletion request. This approach comes at the cost of a large storage overhead.

Guo et al. (2019) define $\epsilon$*-certified removal*, “a very strong theoretical guarantee that a model from which data is removed cannot be distinguished from a model that never observed the data to begin with”, a concept akin to that of differential privacy (Dwork et al. 2006). Combining this with the idea of influence functions (Koh and Liang 2017), they then develop a certified removal mechanism for linear classifiers.

Golatkar et al. (2020a, 2020b) adopt an information theoretic view of unlearning and develop unlearning operations based on linearized model dynamics (drawing inspiration from the *neural tangent kernel* (Jacot et al. 2018; Lee et al. 2019), a technique to describe the gradient descent dynamics of the training of neural networks using kernel methods).

The work done in Guo et al. (2019); Golatkar et al. (2020a, b) may be considered complementary to the method of *linear filtration* we are going to develop in this paper, in the sense that they use strong assumptions (in particular work with linear/linearized models) obtaining stronger guarantees, while linear filtration is an intuitive heuristic, largely agnostic to model architecture.

Sommer et al. (2020) propose a formal framework for verification of machine unlearning, based on machine learning backdoor attacks.

### Membership inference

It is an open problem to find a suitable measure for the quality of unlearning, when employing a heuristic unlearning operation with no or weak theoretical guarantees, i.e. to quantify the remaining influence of “deleted” training data on a model’s parameters. In our experiments we thus take ideas from *membership inference*. The goal of membership inference is to determine whether a given data point has been used in the training process of a given model. A few recent works on membership inference include (Shokri et al. 2017; Yeom et al. 2018; Hayes et al. 2019; Sablayrolles et al. 2019). In particular, our adversarial setup in Sect. 5.1 draws a large amount of inspiration from Shokri et al. (2017), where a binary classifier is trained on the outputs of so-called *shadow models* to decide membership.

### Model inversion

Broadly, *model inversion* may be defined as drawing inferences about private training data from the outputs of a model trained on this data. The term was introduced in Fredrikson et al. (2014). Fredrikson et al. (2015) reconstruct human-recognizable images of individuals from neural networks trained for face recognition, using gradient ascend on the input space. Recent approaches leverage generative adversarial networks (GANs) for model inversion (Zhang et al. 2020).

We remark that model inversion is at its core the result of a correlation between input and output space that is simply captured by the model (and may exist independently of the model), and thus does not necessarily constitute a privacy breach. McSherry (2016) features a highly recommended elaboration of this point in much detail.

Shokri et al. (2017) conclude their discussion of model inversion with the statement that “model inversion produces the average of the features that at best can characterize an entire output class.” Thus, model inversion is of some interest in the specific context of our paper, which focuses on class-wide unlearning (and hence implicitly makes the assumption that a single individual owns all data for an entire output class). We experiment with model inversion in Sect. 5.5, see also Fig. 1.

### Differential privacy

Differential privacy (Dwork et al. 2006; Dwork and Roth 2014; Abadi et al. 2016) limits the influence of individual training points on a model in a precise probabilistic way. We briefly remark that (differential) privacy and data deletion may be considered orthogonal problems, in the sense that private models need not support efficient deletion, and models supporting efficient deletion need not be private. Ginart et al. (2019) discuss this point in additional detail.

## Problem definition

In this section we formalize our notion of unlearning and the threat model we consider.

### Notation

For a vector $v \in {{\,\mathrm{{\mathbb {R}}}\,}}^k$ we use indices ranging from 0 to $(k-1)$ to denote its entries, i.e. $v = (v_0, v_1, \dots v_{k-1})^\top$. One–dimensiona﻿l vector are always column vectors.

For a vector of logits $\ell \in {{\,\mathrm{{\mathbb {R}}}\,}}^k$ let

$$\begin{aligned} \sigma _{i^*}(\ell ) = \frac{\exp ({\ell _{i^*}})}{\sum _{i<k} \exp ({\ell _i})} \in [0,1], \quad \text {for all } i^* < k \end{aligned}$$

and $\sigma (\ell ) = ( \sigma _0(\ell ), \sigma _1(\ell ), \dots , \sigma _{k-1}(\ell ))$. We call $\sigma$ the *softmax function*.

We use uppercase, boldface letters for random variables. If $\mathbf{R}$ is a random variable $P({\mathbf {R}})$ denotes its distribution.

### Classification

We consider a multiclass classification problem: Let ${\mathbf {X}}$ be a random variable taking values in some input space ${\mathcal {X}}$ (e.g. ${\mathcal {X}} = {{\,\mathrm{{\mathbb {R}}}\,}}^{28 \times 28}$), and let ${\mathbf {Y}}$ be a random variable representing class labels taking values in ${\mathcal {Y}} = \{0, 1, \dots , k-1 \}$ for some natural number $k > 2$, with some joint data generating distribution $P({\mathbf {X}}, {\mathbf {Y}})$.

Given $x \in {\mathcal {X}}$, a classifier $h: {\mathcal {X}} \rightarrow {{\,\mathrm{{\mathbb {R}}}\,}}^k$ for this classification problem attempts to estimate logits $\ell = h(x)$ such that

$$\begin{aligned} \sigma (\ell ) \approx P({\mathbf {Y}} \mid {\mathbf {X}} = x). \end{aligned}$$

**Hypothesis class**  In this paper we consider the class ${\mathcal {H}}$ of all classifiers *h* of the form

$$\begin{aligned} h = \mathrm {logistic\ regression} \circ \mathrm {feature\ extraction}, \end{aligned}$$

i.e. classifiers that can be decomposed into a (potentially non-linear) feature extraction followed by a multinomial logistic regression.

**Fig. 2:**
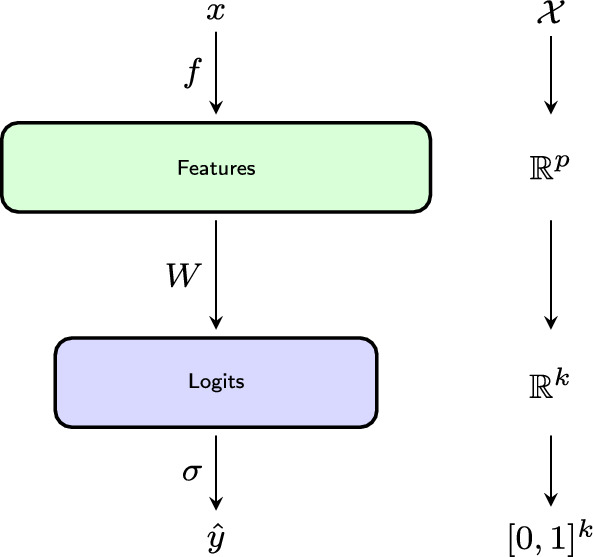
Schematic representation of $\sigma \circ h$, for a classifier $h~=~W \circ f$ in the hypothesis class considered throughout this paper. Here *f* denotes a feature extraction, *W* is a linear transformation, and $\sigma$ is the softmax function. In deep learning terminology “Logits” represents a fully connected layer with *k* units and weights *W*

More formally any $h \in {\mathcal {H}}$ can be expressed as

1 $$\begin{aligned} h: x \mapsto W \cdot f(x) \end{aligned}$$

where $f : {\mathcal {X}} \rightarrow {{\,\mathrm{{\mathbb {R}}}\,}}^p$ denotes the feature extraction, *p* denotes the dimension of the feature space, and *W* is a $(k \times p)$-matrix representing a linear transformation ${{\,\mathrm{{\mathbb {R}}}\,}}^p \rightarrow {{\,\mathrm{{\mathbb {R}}}\,}}^k$. Figure 2 shows a schematic representation of the elements of ${\mathcal {H}}$, where $\sigma$ denotes the softmax function. To simplify notation we do not consider affine transformations, i.e. classifiers of the form $h: x \mapsto W \cdot f(x) + b$ for some $b \in {{\,\mathrm{{\mathbb {R}}}\,}}^k$. However, we remark that our method is easily adapted to this case.

Observe that in particular all deep neural networks for which the output is a densely connected layer with softmax activations fit into the schema discussed in this section.

### Learning algorithms

For a finite training set $D~\subseteq ~{\mathcal {X}}~\times ~{\mathcal {Y}}$, a learning algorithm ${\mathbf {A}}$ calculates a classifier

$$\begin{aligned} h = {\mathbf {A}}(D) \in {\mathcal {H}}. \end{aligned}$$

Note that if ${\mathbf {A}}$ is non-deterministic ${\mathbf {A}}(D)$ can be considered a random variable taking values in ${\mathcal {H}}$.

### Unlearning of classes

Let ${\mathcal {C}} \subsetneq \mathcal Y$ be a set of classes, which we want to unlearn. Consider the multiclass classification problem for the distribution $P({\mathbf {X}}, {\mathbf {Y}} \mid {\mathbf {Y}} \not \in {\mathcal {C}})$, i.e. the original problem with the classes ${\mathcal {C}}$ removed. We define the hypothesis class ${\mathcal {H}}_{\lnot {\mathcal {C}}}$ for this problem similarly to ${\mathcal {H}}$, i.e. $h \in {\mathcal {H}}_{\lnot \mathcal C}$ is of form (1) with *W* a $((k - |{\mathcal {C}}|) \times p)$-Matrix. For $D \subseteq {\mathcal {X}} \times {\mathcal {Y}}$ let $D_{\lnot {\mathcal {C}}} = \{(x,y) \in D : y \not \in {\mathcal {C}} \}$ and let ${\mathbf {A}}_{\lnot {\mathcal {C}}}(D_{\lnot {\mathcal {C}}}) \in {\mathcal {H}}_{\lnot {\mathcal {C}}}$ denote a classifier calculated by some learning algorithm ${\mathbf {A}}_{\lnot \mathcal C}$.

#### Definition 1

(Unlearning) We say that a map

$$\begin{aligned} {\mathfrak {D}}: {\mathcal {H}} \rightarrow {\mathcal {H}}_{\lnot {\mathcal {C}}} \end{aligned}$$

“unlearns ${\mathcal {C}}$ from ${\mathcal {H}}$ with respect to $\mathbf{A}, {\mathbf {A}}_{\lnot {\mathcal {C}}}, D$” if the random variables ${\mathfrak {D}}({\mathbf {A}}(D))$ and ${\mathbf {A}}_{\lnot \mathcal C}(D_{\lnot {\mathcal {C}}})$ have the same distribution over $\mathcal H_{\lnot {\mathcal {C}}}$. We call ${\mathfrak {D}}$ an *unlearning operation* (for ${\mathcal {C}}$).

### Weak unlearning of classes

A good choice of ${\mathfrak {D}}$ will of course depend on the learning algorithm ${\mathbf {A}}$. We mostly concern ourselves with the case where ${\mathbf {A}}$ trains a neural network with a densely connected output layer with softmax activations. Unfortunately it is difficult to understand how a neural network represents knowledge internally (e.g. (Achille et al. 2019)), hence unlearning as defined above may currently be out of reach. We therefore propose a weakening of the above definition.

#### Definition 2

(Weak unlearning) As before let ${\mathfrak {D}}$ be a map ${\mathcal {H}} \rightarrow {\mathcal {H}}_C$ and for ${\mathbf {X}}$ (taking values in the input space ${\mathcal {X}}$ according to the data generating distribution) consider the random variables

$$\begin{aligned} {\mathbf {L}}_{\mathrm {seen}} = h_0({\mathbf {X}}),&\quad \text {where } h_0 = {\mathfrak {D}} ({\mathbf {A}}(D))\\ {\mathbf {L}}_{\lnot \mathrm {seen}}= h_1({\mathbf {X}}),&\quad \text {where } h_1 = {\mathbf {A}}_{\lnot {\mathcal {C}}}(D_{\lnot {\mathcal {C}}}) \end{aligned}$$

i.e. the logit outputs of the respective classifiers, taking values in ${{\,\mathrm{{\mathbb {R}}}\,}}^{k - |{\mathcal {C}}|}$. We say that “${\mathfrak {D}}$ weakly unlearns ${\mathcal {C}}$ from ${\mathcal {H}}$ with respect to ${\mathbf {A}}, {\mathbf {A}}_{\lnot {\mathcal {C}}}, D$” if ${\mathbf {L}}_{\mathrm {seen}}$ and ${\mathbf {L}}_{\lnot \mathrm {seen}}$ have the same distribution over ${{\,\mathrm{{\mathbb {R}}}\,}}^{k - |{\mathcal {C}}|}$. We call ${\mathfrak {D}}$ a *weak unlearning operation* (for ${\mathcal {C}}$).

#### Fact 3

If ${\mathfrak {D}}$ is an unlearning operation, then ${\mathfrak {D}}$ is a weak unlearning operation. $\square$

### Discussion

Definition 1 demands that the distributions over the hypothesis class (i.e. in practical terms the parameter space) are the same, while 2 relaxes this to the distributions over the output space being the same. Intuitively speaking: 1 demands ${\mathfrak {D}}$ make the model *h* “look as if *h* had never seen the data”, while 2 demands ${\mathfrak {D}}$ make the **outputs** of *h* “look as if *h* had never seen the data” We may therefore consider unlearning (in the strong sense) to be a white-box variant and unlearning in the weak sense to be a black-box variant of the definition of unlearning. See section 6 for further discussion.

Abusing the terminology introduced in this section we refer to maps ${\mathfrak {D}}$ that roughly fit definitions 1 and 2 , respectively in the sense that they make the relevant distributions similar in an appropriate divergence measure, as “good”, “satisfactorily performing”, etc. unlearning operations.

#### Threat model

 The considerations about the white- and black-box setting also nicely fit into the common classification of adversaries against machine learning models, as used in adversarial machine learning (e.g., Biggio and Roli (2018); Papernot et al. (2016)). Here, it is common that a white-box adversary has full knowledge of all classifier properties, including training data and weights, while in the black-box scenario, an adversary can either only access the classifier’s final decision or has at most access to the logits. In line with this, considering a white-box adversary makes only sense when evaluating attacks against an unlearning operation in the strong sense (i. e., according to definition 1), while the appropriate mode to evaluate a weak unlearning operation (cf. definition 2) is to consider an adversary who can only access the logit output of the classifier. In general, the white-box setting adheres to Kerckhoffs’ principle (Kerckhoffs 1883) and should be adapted for rigorous theoretical security guarantees. The black-box setting on the other hand, is more akin to practice. The goal of an adversary in our setting is to distinguish between the distributions of the original and unlearned model, given that she has no previous outputs of samples of the deleted class.

## Method

In this section we propose an intuitive weak unlearning operation ${\mathfrak {D}}$ for classes, exploiting the special structure of elements of ${\mathcal {H}}$. In our experiments in section 5 we demonstrate that ${\mathfrak {D}}$ performs satisfactorily for neural networks. We reiterate that the method introduced here is a heuristic, and not an unlearning operation in the strict sense of definition 2.

### Definition of weak unlearning operation ${\mathfrak {D}}_z$

For simplicity of notation let ${\mathcal {C}} = \{0\}$, i.e. we are going to unlearn class 0. However our method easily generalizes to arbitrary ${\mathcal {C}}$. Let $h = W \circ f \in {\mathcal {H}}$ be a classifier. For $j < k$ let

$$\begin{aligned} a_j = {{\,\mathrm{{\mathbb {E}}}\,}}[ h({\mathbf {X}}) \mid {\mathbf {Y}} = j] \in {{\,\mathrm{{\mathbb {R}}}\,}}^{k} \end{aligned}$$

be the expected prediction for class *j*, and let

$$\begin{aligned} A = \Big (a_0 \mid a_1 \mid \dots \mid a_{k-1}\Big ) \in {{\,\mathrm{{\mathbb {R}}}\,}}^{k \times k}. \end{aligned}$$

In practice, we may estimate $a_j$ from the training data. Next, define a map $\pi$ such that for $v = (v_0, v_1, \dots , v_{k-1})^\top \in {{\,\mathrm{{\mathbb {R}}}\,}}^{k}$ we have $\pi (v) = (v_1, v_2, \dots , v_{k-1})^\top$. For arbitrary $z \in {{\,\mathrm{{\mathbb {R}}}\,}}^{(k-1)}$ let

$$\begin{aligned} B_z = \Big (z \mid \pi (a_1) \mid \pi (a_2) \mid \cdots \mid \pi (a_{k-1}) \Big ) \in {{\,\mathrm{{\mathbb {R}}}\,}}^{(k-1) \times k}. \end{aligned}$$

Let $F_z = B_zA^{-1}$ and note that $F_z$ represents the linear transformation which maps the *j*-th row of *A* to the *j*-th row of $B_z$. We call $F_z$ a *filtration matrix*. Let

$$\begin{aligned} W_z = F_z W \in {{\,\mathrm{{\mathbb {R}}}\,}}^{(k-1) \times p}. \end{aligned}$$

Finally, we define a new classifier

$$\begin{aligned} h_z : x \mapsto W_z \cdot f(x). \end{aligned}$$

Our unlearning operation is thus

$$\begin{aligned} {\mathfrak {D}}_z: {\left\{ \begin{array}{ll} {\mathcal {H}} \rightarrow {\mathcal {H}}_{\lnot {\mathcal {C}}}\\ h \mapsto h_z. \end{array}\right. } \end{aligned}$$

We call ${\mathfrak {D}}_z$ a *linear filtration*.

Note that ${\mathfrak {D}}_z$ replaces *W* by $W_z = F_z W$, hence after applying ${\mathfrak {D}}_z$ we may delete *W*. This means that, even though our unlearning operation essentially filters the outputs of the original classifier, the linearity of the filtering operations allows us to absorb the filter into the classifier. This is an important feature in a situation were it may no longer be permissible to store the original classifier. Furthermore, as we consider weak unlearning and thus black-box adversaries with access to the logit output only, in our scenario, an adversary can solely access the final, i.e., filtered, output of the newly derived classifier.

So how do we choose $z \in {{\,\mathrm{{\mathbb {R}}}\,}}^{k-1}$?

#### Naive unlearning

$z = \pi (a_0)$. This gives $F_z = (0 \mid I_{k-1})$, i.e. $F_z = \pi$. Intuitively, we may think of this choice as simply cutting the output unit associated with ${\mathcal {C}}$ out off the classifier. We call this unlearning operation the *naive method* and will use it as a baseline to measure the improvements other methods provide.

#### Normalization

$$\begin{aligned} z = \pi (a_0) - \frac{1}{k-1} \sum _{1 \le i< k} (a_0)_i + \frac{1}{(k-1)^2} \sum _{1 \le i,j < k} (a_j)_i. \end{aligned}$$

**Fig. 3:**
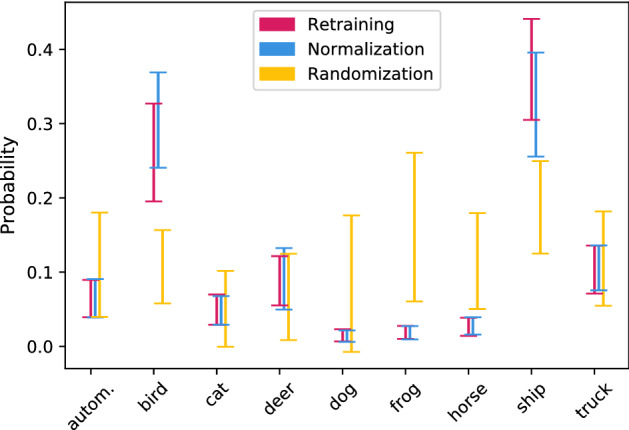
The probability distribution predicted for class “airplane” after its unlearning by either normalization or randomization from models trained on CIFAR-10, compared to models retrained without class “airplane”. The bars are centered around the mean and have length of the standard deviation, over 100 models

This means we shift $\pi (a_0)$ such that its mean becomes the mean of the remaining rows of $B_z$. The intuition behind this choice of *z* is that we would like inputs of the class in ${\mathcal {C}}$ to be misclassified in a “natural” way. We base this method on the assumption that the values in $\pi (a_0)$ encode a natural distribution for predictions of the class in ${\mathcal {C}}$. However, we expect the values in $\pi (a_0)$ to be unnaturally low (absolutely speaking), hence we shift them. We refer to this method as *normalization* or *normalizing filtration*. It is the main method we propose. Figure 3 agrees with the intuition described above: the bars for normalization line up nicely with the bars of the retrained models, while the bars of randomization do not. Proposition 1 summarizes what normalizing filtration achieves.

For comparison we define three more methods.

#### Randomization

$z \sim {\mathcal {N}}(0, I_{k-1})$. We sample *z* from a multivariate normal distribution. We refer to this method as *randomization*.

#### Zeroing

   $z = 0$. We refer to this method as *zeroing*.

#### Transfer

 Instead of retraining the entire model, we only retrain the logistic regression head *W* with the same settings as the original model while holding *f* fixed. We refer to this method as *transfer* unlearning.

##### Proposition 1

(Normalizing filtration.) After applying normalizing filtration to delete class 0 we obtain a classifier $h_z$ with the following property:

$$\begin{aligned} {{\,\mathrm{mean}\,}}({{\,\mathrm{{\mathbb {E}}}\,}}[h_z({\mathbf {X}}) | {\mathbf {Y}} = 0]) = {{\,\mathrm{mean}\,}}({{\,\mathrm{mean}\,}}_{1 \le j < k} (\pi (a_j))). \end{aligned}$$

I.e. the mean of the predictions of inputs belonging to the deleted class is equal to the mean of predictions of the remaining classes.

##### Proof

$$\begin{aligned} {{\,\mathrm{{\mathbb {E}}}\,}}[h_z({\mathbf {X}}) | {\mathbf {Y}} = 0] =&{{\,\mathrm{{\mathbb {E}}}\,}}[W_z f({\mathbf {X}}) | {\mathbf {Y}} = 0]\\ =&{{\,\mathrm{{\mathbb {E}}}\,}}[W_z f({\mathbf {X}}) | {\mathbf {Y}} = 0] \\ =&{{\,\mathrm{{\mathbb {E}}}\,}}[F_z W f({\mathbf {X}}) | {\mathbf {Y}} = 0] \\ =&F_z {{\,\mathrm{{\mathbb {E}}}\,}}[W f({\mathbf {X}}) | {\mathbf {Y}} = 0] \\ =&F_z a_0 = z.\\ \end{aligned}$$

Thus we have:

$$\begin{aligned} {{\,\mathrm{mean}\,}}({{\,\mathrm{{\mathbb {E}}}\,}}[h_z({\mathbf {X}}) | {\mathbf {Y}} = 0]) =&{{\,\mathrm{mean}\,}}(z)\\ =&{{\,\mathrm{mean}\,}}(\pi (a_0)) - \frac{1}{k-1} \sum _{1 \le i< k} (a_0)_i + \frac{1}{(k-1)^2} \sum _{1 \le i,j< k} (a_j)_i \\ =&\frac{1}{(k-1)^2} \sum _{1 \le i,j< k} (a_j)_i\\ =&{{\,\mathrm{mean}\,}}({{\,\mathrm{mean}\,}}_{1 \le j < k} (\pi (a_j))). \end{aligned}$$

$$\square$$

### Computational complexity of ${\mathfrak {D}}_z$

To find ${\mathfrak {D}}_z$ we need to compute the following: **1)** *A*, i.e. the expected predictions for all *k* classes; **2)** $A^{-1}$, the inversion of a $(k \times k)$-Matrix; **3)** *z*, in case *z* has a non-trivial definition (e.g. computing a sample of ${\mathcal {N}}$); **4)** $F_z$, the multiplication of a $((k-1) \times k)$ with a $(k \times k)$-Matrix; **5)** $W_z$, the multiplication of a $((k-1) \times k)$ with a $(k \times p)$-Matrix.

Algorithm 1 lists this computation for the case of deleting class 0. 
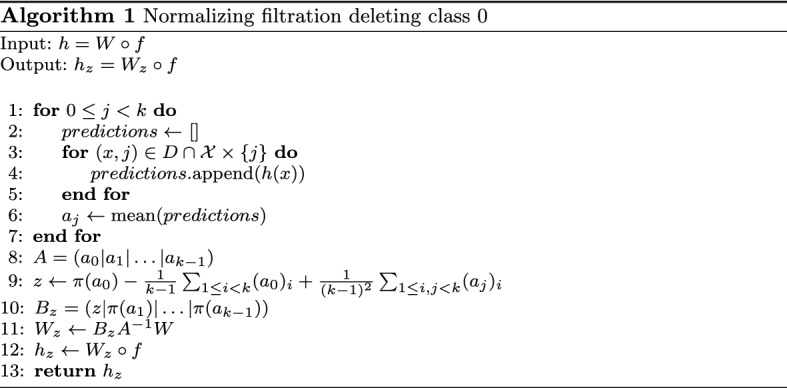


In practice **(1)** will incur the majority of the computational costs, while **(2)**–**(5)** will be negligible. Thus, if we estimate *A* by predicting $\ell$ samples per class the computational complexity of finding ${\mathfrak {D}}_z(h)$ is $\ell \cdot k$ times the complexity of computing a prediction of *h*. We find that ${\mathfrak {D}}_z$ is robust in respect to the quality of the estimation of *A*, hence a small amount of sample points per class suffice, see Sect. 5.4.

Note that the costs incurred by linear filtration are virtually the same for several concurrent deletion requests: we just need to change $B_z$ appropriately, e.g. if we want to delete class 3 in addition to class 0 we need to replace $\pi (a_3)$ in column 3 with some $z_3$. The only additional costs incurred are the computation of $z_3$ (which for our proposed methods are negligible).

## Experiments

### Evaluation method

By definition 2 the quality of a weak unlearning operation depends on the similarity of the resulting distributions $P({\mathbf {L}}_{\mathrm {seen}})$ and $P({\mathbf {L}}_{\lnot \mathrm {seen}})$. We begin by defining a divergence measure for distributions based on the Bayes error rate. This then motivates us to empirically evaluate the performance of the unlearning operations proposed in Sect. 4 by training binary classifiers on the pre-softmax outputs of our models.

#### Classifier advantage

 Let ${\mathbf {B}}$ be a Bernoulli random variable uniformly taking values in $\{0,1\}$. Let

$$\begin{aligned} \mathbf{U} = {\mathbf {L}}_{\mathrm {seen}} \cdot (1 - {\mathbf {B}}) + \mathbf{L}_{\lnot \mathrm {seen}} \cdot {\mathbf {B}} \end{aligned}$$

be the mixture of $\mathbf{L}_{\mathrm {seen}}$ and ${\mathbf {L}}_{\lnot \mathrm {seen}}$. Let

$$\begin{aligned} b : {{\,\mathrm{dom}\,}}({\mathbf {U}}) \rightarrow \{0,1\} \end{aligned}$$

be a binary classifier operating on the mixture ${\mathbf {U}}$ and define

2 $$\begin{aligned} \alpha _{b} = 2 \big ( {{\,\mathrm{{\mathbb {E}}}\,}}[ P({\mathbf {B}} = b({\mathbf {U}}) \mid {\mathbf {U}}) ] - \frac{1}{2} \big ). \end{aligned}$$

We call $\alpha _{b}$ the *classifier advantage* of *b*. Intuitively speaking, $\alpha _b$ is a measure for how good *b* is at telling the mixture ${\mathbf {U}}$ apart. Let

$$\begin{aligned} b^*: u \mapsto {{\,\mathrm{argmax}\,}}_{i < 2} P({\mathbf {B}} = i \mid {\mathbf {U}} = u) \end{aligned}$$

be the Bayes optimal classifier for $P({\mathbf {U}}, {\mathbf {B}})$ Then $\alpha _{b^*} \in [0,1]$ and it is a measure for the difference between $P({\mathbf {L}}_{\mathrm {seen}})$ and $P({\mathbf {L}}_{\lnot \mathrm {seen}})$, based on how much better the Bayes optimal classifier $b^*$ performs than random guessing. A value of $\alpha _{b^*}$ close to 0 indicates that $P(\mathbf{L}_{\mathrm {seen}})$ and $P({\mathbf {L}}_{\lnot \mathrm {seen}})$ are similar.

#### Experimental setup

 Assume that $b^*$ can be approximated sufficiently well by a classifier *b* derived via a state-of-the-art binary classification algorithm. Then $\alpha _b$ approximates $\alpha _{b^*}$, hence we consider a low value of $\alpha _b$ to be evidence for the similarity of the distributions of $\mathbf{L}_{\mathrm {seen}}$ and ${\mathbf {L}}_{\lnot \mathrm {seen}}$. Note that even if we do not believe that *b* approximates $b^*$ well, we may still use $\alpha _b$ as a relative performance measure for different unlearning operations.

Drawing i.i.d. samples from $P({\mathbf {L}}_{\mathrm {seen}})$ and $P({\mathbf {L}}_{\lnot \mathrm {seen}})$ is computationally expensive as it requires us to run the algorithms ${\mathbf {A}}(D)$ respectively ${\mathbf {A}}_{\lnot {\mathcal {C}}}(D_{\lnot {\mathcal {C}}})$ for every sample point. In our experiments we thus take a pragmatic approach. We train 100 models which get to see the full training data and 100 models which get to see the training data with ${\mathcal {C}}$ removed, i.e. models that unlearned by retraining from scratch. We then apply our unlearning operation to each of the models that got to see the full training data. For every single model we then calculate the predictions for the full test data by that model, without applying the softmax activations of the output layer. We label the predictions made by models that originally got to see the full training data with **“seen”** and predictions made by models which never got to see the training data for ${\mathcal {C}}$ as **“not seen”**.

**Fig. 4:**
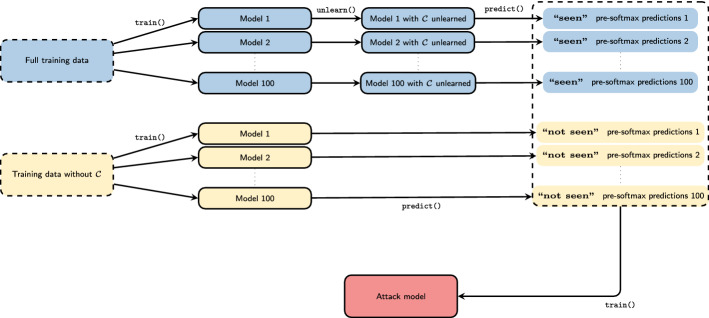
Experimental setup: On the full training data we train 100 models by ${\mathbf {A}} =$train(). To these models we then apply an unlearning operation ${\mathfrak {D}} =$unlearn(). We then predict() our test data for each of these models and label these predictions **“seen”**. On the training data with ${\mathcal {C}}$ removed we train 100 models by ${\mathbf {A}}_{\lnot {\mathcal {C}}} =$train(). We then predict() our test data for each of these models and label these predictions **“not seen”**. Finally, we use all labeled predictions as the training/test data of a binary classifier *b*, which we employ as our “attack model”. We interpret low test accuracy of *b* as evidence for good performance of a weak unlearning operation

Finally, we train a binary classifier *b* that given a prediction attempts to predict its label **“seen”** or **“not seen”**. For this purpose we use the predictions of 70 models of either category as training data and the predictions of the remaining 30 models of either category as test data. Figure 4 shows a schematic representation of our setup. In practice, we train a separate binary classifier on the predictions of each class.

Note the subtle difference of our setup to the shadow model setup of (Shokri et al. 2017), where the authors ask their binary classifier (there “attack model”): “Do these outputs look like they come from a member of the training set?”, and hope for an accurate answer, such that their membership inference attack succeeds. We ask our binary classifier: “Do these outputs look like they come from a model that has seen ${\mathcal {C}}$?”, and hope for an inaccurate answer, as we hope that our unlearning operation prevents the attack.

### Data

#### MNIST

 The MNIST dataset (Lecun et al. 1998) contains 70, 000 $28{\times }28$ images of handwritten digits in 10 classes, with 7, 000 images per class, split into 60, 000 training and 10, 000 test images.

#### CIFAR-10

The CIFAR-10 dataset (Krizhevsky 2009) contains 60, 000 $32{\times }32{\times }3$ images in 10 classes, with 6, 000 images per class, split into 50, 000 training and 10, 000 test images.

#### AT&T Faces

 The AT&T Laboratories Cambridge Database of Faces (AT&T Laboratories Cambridge 1994) contains 400 $92{\times }112$ images of 40 subjects, with 10 images per subject.

### Network architectures

#### MLP

For the MNIST dataset, we evaluate our unlearning method on a multilayer perceptron (MLP), with one hidden layer of 50 units.

#### CNN

For the CIFAR-10 dataset, we evaluate our unlearning method on convolutional neural networks (CNNs). Our networks consist of two convolutional (with 16 respectively 32 $3{\times }3$ filters) and two max-pooling layers, followed by a fully connected layer with *p* units with rectified linear activations and a softmax output layer. We experiment with $p \in \{64,256,1024 \}$. Note that our unlearning operation works by manipulating $W \in {{\,\mathrm{{\mathbb {R}}}\,}}^{k \times p}$, thus we want to investigate how the size of *W* affects unlearning performance.

#### ResNet

For the AT&T faces dataset we evaluate our unlearning method on a residual neural network architecture (He et al. 2016). We use a convolutional layer (with 8 $5{\times }5$ filters), followed by 5 downsampling residual blocks (with $2^{i+3}$ $3{\times }3$ filters in the *i*-th block), followed by global max-pooling and a softmax output layer.

### Results

We experiment with the following classification algorithms: nearest neighbors (NN), random forests (RF), and AdaBoost (AB). For the models trained on MNIST and CIFAR-10 we unlearn the first class (of 10) in the dataset (the digit “zero” and the class “airplane”, respectively). For the models trained on AT&T Faces we unlearn the first 4 individuals (of 40) in the dataset.

Table 1 shows classifier advantages (remember equation (2)) for the MLPs trained on MNIST. We observe a significant decrease of advantage when unlearning by normalization compared to the naive method for the unlearned class, and similar advantage for the remaining classes. The classifier advantage for the transfer learning approach is between the values for naive unlearning and normalization for the unlearned class, and considerably higher for the remaining class. Table 2 shows classifier advantages for the CNNs trained on CIFAR-10 (with $p=256$). We observe a vast decrease of advantage when unlearning by normalization compared to the naive method for the unlearned class, while randomization and zeroing do not provide such benefits. For the transfer learning approach, the classifier advantages are high for both unlearning and remaining classes. Table 3 shows classifier advantages for the residual networks trained on AT&T Faces. We observe a slight decrease of advantage when unlearning by normalization compared to the naive method for the unlearned classes, and similar advantage for the remaining classes. Again, for the transfer learning approach, the classifier advantages are high for both unlearning and remaining classes.

**Table 1:** Classifier advantage for 1 unlearned and 9 remaining classes, for MLPs trained on MNIST

Unlearned	NN	RF	AB
Naive	0.593	0.609	0.641
Normalization	0.327	0.362	0.438
Transfer	0.462	0.552	0.550
Remaining	NN	RF	AB
Naive	0.048	0.090	0.098
Normalization	0.041	0.089	0.095
Transfer	0.315	0.383	0.428

**Table 2:** Classifier advantage for 1 unlearned and 9 remaining classes, for CNNs trained on CIFAR-10, with $p = 256$

Unlearned	NN	RF	AB
Naive	0.609	0.457	0.590
Normalization	0.146	0.110	0.093
Randomization	0.634	0.579	0.582
Zeroing	0.642	0.566	0.575
Transfer	0.951	0.840	0.952
Remaining	NN	RF	AB
Naive	0.115	0.080	0.109
Normalization	0.148	0.097	0.118
Randomization	0.416	0.279	0.230
Zeroing	0.421	0.276	0.219
Transfer	0.949	0.819	0.948

**Table 3:** Classifier advantage for 4 unlearned and 36 remaining classes, for residual networks trained on AT&T faces

Unlearned	NN	RF	AB
Naive	0.467	0.573	0.574
Normalization	0.381	0.454	0.462
Transfer	0.985	0.977	0.978
Remaining	NN	RF	AB
Naive	0.149	0.266	0.245
Normalization	0.152	0.263	0.246
Transfer	0.998	0.996	0.996

#### Effect of sample size

We investigate the effect of sample size for estimating the matrix of mean predictions *A* (recall Sect. 4.1). Table 4 shows the results for CNNs trained on CIFAR-10 (with $p = 256$). Unlearning by normalization, compared to the naive method, we observe a strong decrease of advantage for the unlearned class for a sample size of 10 per class ($1\%$ of the test data), and for a sample size of 100 per class ($10\%$ of the test data) we observe performance comparable to the estimation based on the full test data.

**Table 4:** Classifier advantage for CNNs trained on CIFAR-10, for different sample size *s* per class, with $p=256$

Unlearned	*s*	NN	RF	AB
Naive	–	0.609	0.457	0.590
Normalization	10	0.193	0.114	0.122
Normalization	100	0.156	0.095	0.111
Normalization	1000	0.146	0.110	0.093
Remaining	*s*	NN	RF	AB
Naive	–	0.115	0.080	0.109
Normalization	10	0.205	0.142	0.171
Normalization	100	0.169	0.108	0.136
Normalization	1000	0.148	0.097	0.118

#### Effect of p

For the CNNs trained on CIFAR-10 we investigate the effect of the number *p* of units in the fully connected layer. Table 5 shows that unlearning by normalization significantly decreases classifier advantage for all tested values of *p* on the unlearned class. We observe a slight increase of advantage for the remaining classes, that appears to get slightly worse with *p* increasing.

**Table 5:** Classifier advantage, for CNNs trained on CIFAR-10, for different numbers *p* of units in the fully connected layer

Unlearned	*p*	NN	RF	AB
Naive	64	0.290	0.321	0.380
Normalization	64	0.086	0.106	0.119
Naive	256	0.609	0.457	0.590
Normalization	256	0.146	0.110	0.093
Naive	1024	0.627	0.485	0.603
Normalization	1024	0.174	0.138	0.138
Remaining	*p*	NN	RF	AB
Naive	64	0.040	0.043	0.044
Normalization	64	0.044	0.040	0.045
Naive	256	0.115	0.080	0.109
Normalization	256	0.148	0.097	0.118
Naive	1024	0.129	0.090	0.115
Normalization	1024	0.174	0.104	0.129

#### Effect on classification accuracy

While implicit in definition 2, an important consideration for the design of an unlearning operation is that we do not want to decrease the performance of a classifier on the remaining classes. Table 6 reports the test images for which the most likely label predicted was changed by one of our unlearning methods, compared to the naive method. Normalization did not change any labels (and thus in particular non of the correct ones).

**Table 6:** The amount of labels changed in percent, when compared to the naive method, for 100 models trained on CIFAR-10, and $p = 256$. The “all”-column reports the value for predictions of the entire test set, the “unl.”-column for predictions of images of the unlearned class, and the “cor.”-column for predictions of images of the remaining classes that are correctly predicted by the naive method. The “acc.”-column shows the mean classification accuracy

Deletion method	All	Unl.	Cor.	Acc
Naive	–	–	–	69.2
Normalization	0.0	0.0	0.0	69.2
Randomization	20.1	48.8	11.8	64.8
Zeroing	18.3	45.4	10.3	65.7

#### Summary of experimental results

Normalization (1) decreased classifier advantage on the unlearned classes in all three experiments; (2) showed robustness with regards to sample size for parameter estimation; (3) performed well for different dimensions of the feature space, and (4) did not negatively affect classification accuracy. The transfer learning approach performed poorly for all three datasets.

### Model inversion

Figures 1 (on page 2) and 5 show the results of a “model inversion attack”, i.e. gradient ascend on the input space for a model trained on AT&T Database of Faces. We use a neural network consisting of two fully connected layers with 1000 and 300 units respectively with sigmoid activations and a softmax output layer.

Visually, naive unlearning barely affects the quality of the reconstruction for any of the classes (in particular not the unlearned class). On the other hand the normalization method greatly disturbs the reconstruction of the unlearned class, while barely affecting the remaining classes. In accordance with our discussion of model inversion in Sect. 2, we thus interpret Fig. 1 as visual evidence suggesting a desirable effect of our normalization method on the correlation between input and output space represented by our model. We leave a more detailed investigation of this phenomenon for future work.

### Random direction Kolmogorov–Smirnov statistics

Given $\varPhi \subseteq {{\,\mathrm{{\mathbb {R}}}\,}}^{k - |{\mathcal {C}}|}$, and having drawn samples $S_1$, $S_2$ from $P({\mathbf {L}}_{\mathrm {seen}})$ and $P({\mathbf {L}}_{\lnot \mathrm {seen}})$ respectively in the fashion described in Sect. 5.1, we compute the statistic

3 $$\begin{aligned} {{\,\mathrm{KS}\,}}_\varPhi (S_1, S_2) = \frac{1}{|\varPhi |} \sum _{\phi \in \varPhi } {{\,\mathrm{KS}\,}}_{\text {two sample}}(\phi \cdot S_1, \phi \cdot S_2). \end{aligned}$$

Here $KS_{\text {two sample}}$ denotes the two-sample Kolmogorov-Smirnov statistic and $\phi \cdot S$ is shorthand for $\{\phi \cdot s : s \in S \} \subseteq {{\,\mathrm{{\mathbb {R}}}\,}}$.

Table 7 reports the ${{\,\mathrm{KS}\,}}_\phi$ statistic for $\varPhi$ consisting of 1000 unit vectors pointing in uniform random directions. We compare naive unlearning to normalizing filtration for the CIFAR-10 experiment employing CNNs ($p = 256$), and the MNIST experiment employing MLPs. As noted in Sect. 5.1 the samples we draw are not i.i.d., hence we also report a baseline ${{\,\mathrm{KS}\,}}_\phi$ statistic computed from two independent batches of 100 models trained without the class we unlearn.

The results paint a picture similar to Sect. 5.4. In both experiments normalizing filtration decreases ${{\,\mathrm{KS}\,}}_\varPhi$ for the unlearned class, with somewhat better performance in the CIFAR-10 experiment, while the ${{\,\mathrm{KS}\,}}_\varPhi$ remains unchanged for the remaining classes.

**Table 7:** ${{\,\mathrm{KS}\,}}_\varPhi$ for $\varPhi$ consisting of 1000 unit vectors pointing in uniform random directions. (Lower values are better.)

Unlearned	CIFAR-10	MNIST
Naive	0.115	0.184
Normalization	0.038	0.1
Baseline	0.015	0.017
Remaining	CIFAR-10	MNIST
Naive	0.037	0.034
Normalization	0.038	0.034
Baseline	0.013	0.023

## Discussion

In Sect. 3 we made a didactic choice to introduce the special hypothesis class ${\mathcal {H}}$ (which permitted absorption of the filtration operation) before defining unlearning. It should however be clear how definitions 1 and 2 are applicable to any class of classifiers, and in the somewhat more common setting of deletion requests of single data points. We would like to emphasize our belief that in the light of the non-deterministic nature of many learning algorithms a probabilistic definition of unlearning, such as chosen in (Ginart et al. 2019) (there “deletion”) and our work, is necessary.

**Fig. 5:**
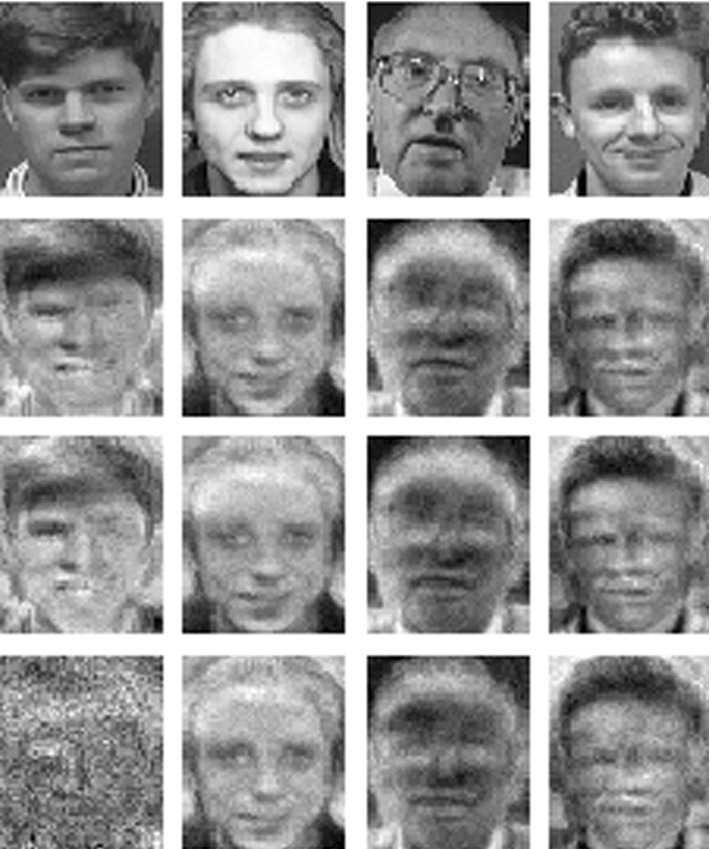
Model inversion for a toy model trained on the AT&T Faces dataset with 4 classes. The top row shows one training image of each class, the second row reconstructions of classes by model inversion, the third row reconstructions after naive unlearning of the class in the first column, the bottom row reconstructions after unlearning the class in the first column by *normalizing filtration*. See Fig. 6 for the remaining classes

For contrast let us consider the definition of unlearning in (Bourtoule et al. 2019) that asks to find a model that “could have been obtained” without looking at the deleted training data. Since all models on discrete digital systems necessarily have a finite parameter space we very much could obtain any model without looking at any data by guessing its parameters. What happened here?

Guessing a model’s parameters can be considered drawing a sample from a uniform distribution over the parameter space. On the other hand a probabilistic definition such as 1 requires the distribution over the parameter space to be the same as if we had run the original learning algorithm without using the deleted data, which for reasonable learning algorithms is certainly not uniform. If we would like to stick to an informal definition we should therefore say that a model “could have been obtained, with reasonable likelihood”.

We further conclude that it is an important consideration whether a definition of unlearning makes sense when not read in a benevolent way (e.g. by a party whose interest in unlearning stems from of legal obligation). In fact our definition of weak unlearning 2, i.e. unlearning in a black-box sense, suffers from a similar issue. A malicious way to define a weak unlearning operations ${\mathfrak {D}}$ is the following: for any classifier *h*, simply train a new classifier $h'$ without using the deleted data, then define ${\mathfrak {D}}(h) = h'' = (h,h')$, where $h''(x) = h'(x)$. The outputs of ${\mathfrak {D}}(h)$ look exactly like those of $h'$, thus ${\mathfrak {D}}$ is indeed an unlearning operation in the black-box sense, however we never actually deleted *h*. Let us thus emphasize that the weak definition of unlearning is only applicable when acting in good faith.

**Fig. 6:**
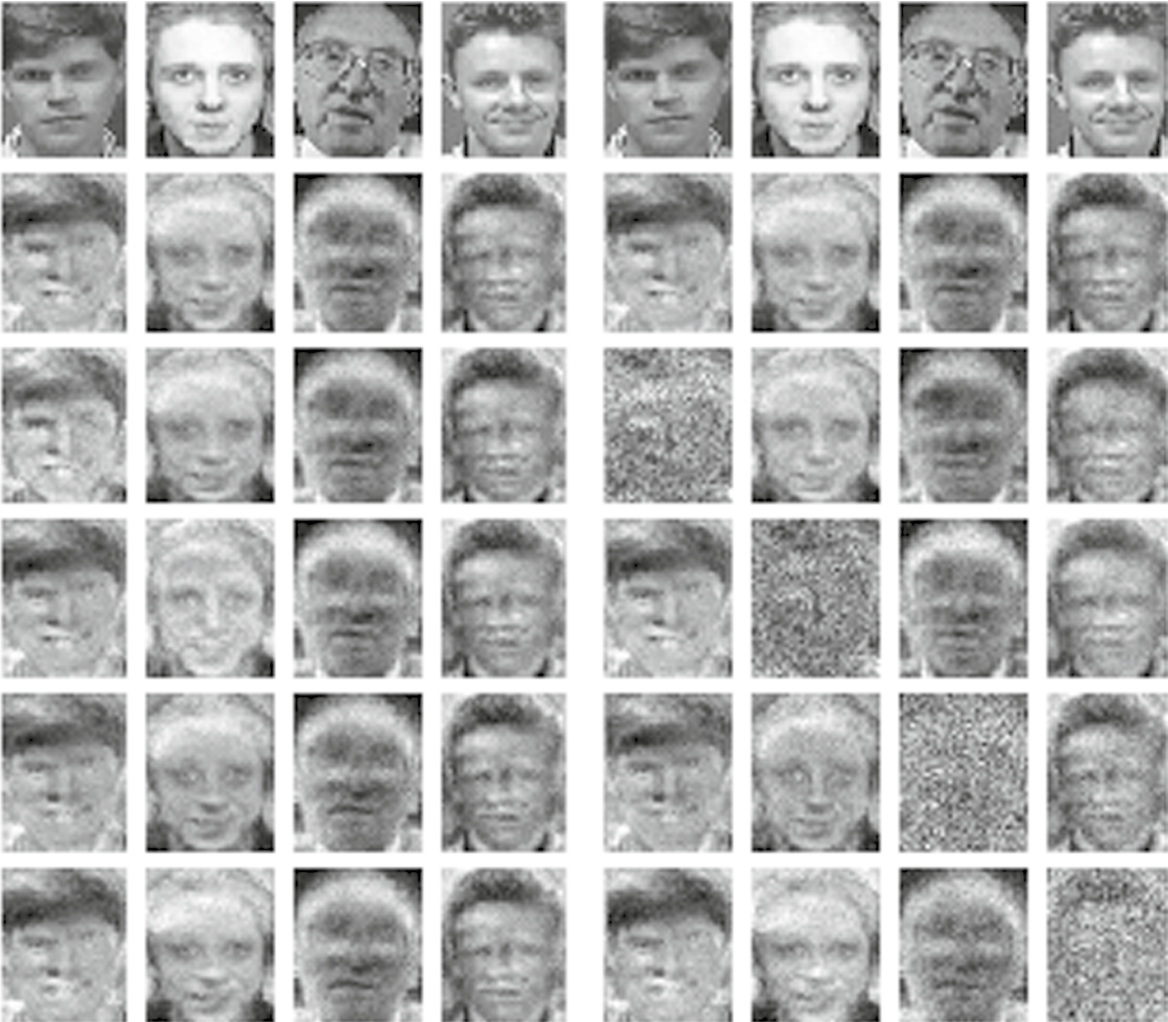
Model inversion for a toy model trained on the AT&T Faces dataset with 4 classes. On the left side we show results for naive unlearning, on the right side for *normalizing filtration*. On either side: The top row shows one training image of each class, the second row reconstructions of classes by model inversion, the $(i + 2)$-th row shows the reconstructions after unlearning the class in the *i*-th column by the respective unlearning operation

## Conclusion

We considered the problem of unlearning in a class-wide setting, for classifiers predicting logits. We developed *normalizing filtration* as an unlearning method, with compelling visual results (figure 6). These are backed up by good experimental results with regards to to our proposed definition of *weak unlearning* and our metrics of *classifier advantage* (2) and the ${{\,\mathrm{KS}\,}}_\varPhi$ statistic (3). We emphasize once again the black-box nature of weak unlearning, entailing the black-box nature of our proposed unlearning operation. While linear filtration can be absorbed into the final layer of a classifier (for the hypothesis class considered), our approach remains somewhat limited with regards to its shallowness. In future work we hope to find methods that allow for deeper absorption, thus hopefully leading to stronger privacy guarantees. On the other hand our method’s intuitive simplicity nicely complements concurrent approaches such as (Guo et al. 2019; Golatkar et al. 2020a, b). Another promising direction may be to enhance our method by some form of shrinkage of the logits, in case the unlearned class constitutes a large part of the misclassifications of one of the remaining classes and plan to explore the connection to the field of private prediction van der Maaten and Hannun (2020). Finally it should be noted that, while the “right to be forgotten” inspired our research, whether our approach is adequate in this context is for legal scholars to decide.

## Data Availability

We only use data that is openly available.

## Appendix

### A more complete versions of tables from section 5.4

#### MNIST – MLP experiment

See Tables 8, 9.

**Table 8:** Accuracy (in percent) and cross-entropy loss, mean ± standard deviation, for 100 MLPs trained on MNIST. *p* is the number of units in the fully connected layer, i.e. the dimension of the feature space. *s* is the sample size per class, used to estimate the mean predictions

Unlearned	NN	RF	AB
Naive	0.593	0.609	0.641
Normalization	0.327	0.362	0.438
Randomization	0.910	0.869	0.902
Zeroing	0.919	0.886	0.909
Remaining	NN	RF	AB
Naive	0.048	0.090	0.098
Normalization	0.041	0.089	0.095
Randomization	0.116	0.132	0.133
Zeroing	0.119	0.129	0.132

**Table 9:** Classifier advantage for 1 unlearned and 9 remaining classes, for MLPs trained on MNIST. *p* is the number of units in the fully connected layer, i.e. the dimension of the feature space. *s* is the sample size per class, used to estimate the mean predictions

	Accuracy	Loss
Before unlearning	97.1 ± 0.19	0.10 ± 0.01
Baseline model	97.1 ± 0.18	0.09 ± 0.01
Naive	97.1 ± 0.20	0.10 ± 0.01
Normalization	97.1 ± 0.20	0.10 ± 0.01
Randomization	96.7 ± 0.33	0.11 ± 0.01
Zeroing	96.7 ± 0.30	0.11 ± 0.01

#### CIFAR10 – CNN experiment

Tables 10, 11.

**Table 10:** Classifier advantage for 1 unlearned and 9 remaining classes, for CNNs trained on CIFAR-10. *p* is the number of units in the fully connected layer, i.e. the dimension of the feature space. *s* is the sample size per class, used to estimate the mean predictions

Unlearned	*p*	*s*	NN	RF	AB
Naive	64	–	0.290	0.331	0.380
Normalization	64	10	0.113	0.135	0.137
Normalization	64	100	0.089	0.105	0.129
Normalization	64	–	0.086	0.106	0.119
Randomization	64	–	0.547	0.487	0.528
Zeroing	64	–	0.599	0.534	0.560
Naive	256	–	0.609	0.457	0.590
Normalization	256	10	0.193	0.114	0.122
Normalization	256	100	0.156	0.095	0.111
Normalization	256	–	0.146	0.110	0.093
Randomization	256	–	0.634	0.579	0.582
Zeroing	256	–	0.642	0.566	0.575
Naive	1024	–	0.627	0.485	0.603
Normalization	1024	10	0.230	0.164	0.179
Normalization	1024	100	0.216	0.157	0.172
Normalization	1024	–	0.174	0.138	0.138
Randomization	1024	–	0.741	0.637	0.682
Zeroing	1024	–	0.746	0.642	0.680
*Remaining*					
Naive	64	–	0.040	0.043	0.044
Normalization	64	10	0.072	0.075	0.080
Normalization	64	100	0.047	0.044	0.047
Normalization	64	–	0.044	0.040	0.045
Randomization	64	–	0.240	0.203	0.174
Zeroing	64	–	0.246	0.204	0.163
Naive	256	–	0.115	0.080	0.109
Normalization	256	10	0.205	0.142	0.171
Normalization	256	100	0.169	0.108	0.136
Normalization	256	–	0.148	0.097	0.118
Randomization	256	–	0.416	0.279	0.230
Zeroing	256	–	0.421	0.276	0.219
Naive	1024	–	0.129	0.090	0.115
Normalization	1024	10	0.242	0.149	0.182
Normalization	1024	100	0.217	0.128	0.146
Normalization	1024	–	0.174	0.097	0.118
Randomization	1024	–	0.506	0.309	0.249
Zeroing	1024	–	0.508	0.310	0.251

**Table 11:** Accuracy (in percent) and cross-entropy loss, mean ± standard deviation, for 100 CNNs trained on CIFAR10. *p* is the number of units in the fully connected layer, i.e. the dimension of the feature space. *s* is the sample size per class, used to estimate the mean predictions

	*p*	*s*	Accuracy	Loss
Before unlearning	64	–	65.1 ± 1.01	1.01 ± 0.03
Retraining	64	–	66.0 ± 1.06	0.96 ± 0.03
Naive	64	–	66.4 ± 1.04	0.95 ± 0.03
Normalization	64	10	66.4 ± 1.04	0.95 ± 0.03
Normalization	64	100	66.4 ± 1.04	0.95 ± 0.03
Normalization	64	–	66.4 ± 1.04	0.95 ± 0.03
Randomization	64	–	61.1 ± 2.08	1.17 ± 0.10
Zeroing	64	–	62.3 ± 1.76	1.12 ± 0.09
Before unlearning	256	–	68.1 ± 0.85	0.93 ± 0.02
Retraining	256	–	69.2 ± 0.80	0.89 ± 0.02
Naive	256	–	69.2 ± 0.83	0.88 ± 0.02
Normalization	256	10	69.2 ± 0.83	0.88 ± 0.02
Normalization	256	100	69.2 ± 0.83	0.88 ± 0.02
Normalization	256	–	69.2 ± 0.83	0.88 ± 0.02
Randomization	256	–	64.9 ± 1.76	1.07 ± 0.08
Zeroing	256	–	65.7 ± 1.40	1.03 ± 0.06
Before unlearning	1024	–	69.7 ± 0.81	0.93 ± 0.03
Retraining	1024	–	70.5 ± 0.82	0.88 ± 0.03
Naive	1024	–	70.7 ± 0.82	0.88 ± 0.03
Normalization	1024	10	70.7 ± 0.82	0.88 ± 0.03
Normalization	1024	100	70.7 ± 0.82	0.88 ± 0.03
Normalization	1024	–	70.7 ± 0.82	0.88 ± 0.03
Randomization	1024	–	67.1 ± 1.55	1.06 ± 0.08
Zeroing	1024	–	67.6 ± 1.16	1.03 ± 0.05

## Code availability

We will make the code available at Github upon acceptance of the manuscript.
